# Isolation of non-tuberculous mycobacteria from pastoral ecosystems of Uganda: Public Health significance

**DOI:** 10.1186/1471-2458-11-320

**Published:** 2011-05-16

**Authors:** Clovice Kankya, Adrian Muwonge, Berit Djønne, Musso Munyeme, John Opuda-Asibo, Eystein Skjerve, James Oloya, Vigdis Edvardsen, Tone B Johansen

**Affiliations:** 1 Department of Veterinary Public Health, School of Veterinary Medicine, Makerere University, P.O. Box 7062, Kampala, Uganda; 2 Centre for Epidemiology and Biostatistics, Norwegian School of Veterinary Science, P.O. Box 8146 Dep., 0033 Oslo, Norway; 3 Norwegian Veterinary Institute, P.O. Box 750, N-0106 Oslo, Norway; 4 Department of Disease Control, School of Veterinary Medicine, University of Zambia, P.O. Box 32379, Lusaka, Zambia

## Abstract

**Background:**

The importance of non-tuberculous mycobacteria (NTM) infections in humans and animals in sub-Saharan Africa at the human-environment-livestock-wildlife interface has recently received increased attention. NTM are environmental opportunistic pathogens of humans and animals. Recent studies in pastoral ecosystems of Uganda detected NTM in humans with cervical lymphadenitis and cattle with lesions compatible with bovine tuberculosis. However, little is known about the source of these mycobacteria in Uganda. The aim of this study was to isolate and identify NTM in the environment of pastoral communities in Uganda, as well as assess the potential risk factors and the public health significance of NTM in these ecosystems.

**Method:**

A total of 310 samples (soil, water and faecal from cattle and pigs) were examined for mycobacteria. Isolates were identified by the INNO-Lipa test and by 16S rDNA sequencing. Additionally, a questionnaire survey involving 231 pastoralists was conducted during sample collection. Data were analysed using descriptive statistics followed by a multivariable logistic regression analysis.

**Results:**

Forty-eight isolates of NTM were detected; 25.3% of soil samples, 11.8% of water and 9.1% from animal faecal samples contained mycobacteria. Soils around water sources were the most contaminated with NTM (29.8%). Of these samples, *M. fortuitum-peregrinum *complex, *M. avium *complex, *M. gordonae*, and *M. nonchromogenicum *were the most frequently detected mycobacteria. Drinking untreated compared to treated water (OR = 33), use of valley dam versus stream water for drinking and other domestic use (OR = 20), sharing of water sources with wild primates compared to antelopes (OR = 4.6), sharing of water sources with domestic animals (OR = 5.3), and close contact with cattle or other domestic animals (OR = 13.8) were the most plausible risk factors for humans to come in contact with NTM in the environment.

**Conclusions:**

The study detected a wide range of potentially pathogenic NTM from the environment around the pastoral communities in Uganda. Drinking untreated water and living in close contact with cattle or other domestic animals may be risk factors associated with the possibility of humans and animals acquiring NTM infections from these ecosystems.

## Background

Although members of the *Mycobacterium tuberculosis *complex (MTC) are responsible for the majority of mycobacterial infections worldwide, environmental opportunistic infections due to non-tuberculous mycobacteria (NTM) are increasingly becoming more of a public health challenge [1]. Globally, the picture of NTM infections has drastically changed with the emergence of the HIV/AIDS pandemic, due to the direct consequence of the immunosuppression [2,3]. Synonyms for the NTM group of mycobacteria are atypical mycobacteria or mycobacteria other than tuberculosis (MOTT) [4,5]. They include both slow growing mycobacteria (SGM) where colony formation requires at least seven days and rapid growing mycobacteria (RGM) forming colonies in less than seven days [1,6]. The most important potentially pathogenic mycobacteria are *M. avium*, *M. intracellulare*, *M. kansasii, M. xenopi*, and *M. abscessus *[2,7]. In addition, an increasing number of NTM previously considered non-pathogenic, have been shown to cause infections in animals and humans [8,9].

Exposure to NTM has different public health implications [10,11], as they are capable of causing pulmonary disease, disseminated disease or localized lesions in both immunocompetent and immunocompromised individuals [12]. NTM can also colonise individuals without developing disease [13], and they have been known to induce non-specific immune response and thereby lead to false positive reactions in tuberculin testing [14-16]. To be able to determine the level and prevalence of NTM infections, it is important to identify the mycobacterial species involved in infections. This has traditionally been addressed by culture and biochemical methods, but genetic methods are increasingly being used for detection, identification and characterisation. Hybridisation assays and sequencing of genes coding for ribosomal RNA are frequently used for identification of NTM [1]. In developing countries, it may be difficult to assess the prevalence of NTM infections, mainly due to the fact that identification of the species involved is generally not done, and these diseases are often under-diagnosed or misdiagnosed as tuberculosis.

The majority of NTM are opportunistic pathogens, and true inhabitants of the environment found as saprophytes, commensals and symbionts [17] in ecosystems shared between humans and animals [2,14,18]. Soil and natural open water sources are known to contain mycobacteria, and play a key role as sources for human and animal infections [1]. Like in other developing countries, the communities and their animals in the Ugandan pastoral ecosystems face challenges with safe drinking water. They rely mainly on untreated water from open natural sources as valley tanks, dams, streams, and swamps for drinking and household use [19], although in some areas water from boreholes is available. The limited water sources are shared between humans, their livestock and wildlife. Few reports have investigated the distribution of NTM in the pastoral environment, although NTM have been isolated from cattle carcasses and humans with lymphadenitis in different countries including Uganda [20,21]. The environment is the most likely reservoir for these infections, as there is no evidence of human-to-human or animal-to-human transmission [22,23].

Recently, NTM infections in humans and animals in the sub-Saharan Africa have received increasing attention, due to reports that respiratory infections, including mycobacterial infections, ranked high in Uganda (18.2%), and that the prevalence of HIV/AIDS is found to be as high as 7% [24-26]. In addition a "One health, One ecosystem" study was carried out in Uganda, detecting NTM from humans with cervical lymphadenitis and cattle with lesions compatible with bovine tuberculosis [20,21]. This demonstrates an increasing role for NTM as opportunistic pathogens in both immune-competent and immune-compromised individuals [12]. As the source of these infections is not known, there is a clear need for linking data from bacteriological culturing and characterisation to epidemiological studies to allow more inference about the real sources of NTM infections in the region.

The aim of this study was to contribute to bridging this gap of knowledge by isolating and identifying NTM from the environment of the pastoral communities in Uganda, as well as identify potential factors associated with detection of NTM and assess the possible public health significance.

## Methods

### Study areas and selection

Mubende and Nakasongola districts located in central Uganda represent two important traditional pastoral areas, typical of the Ugandan pastoral ecosystem. The two were selected for the study due to their conservative traditional pastoral practices such as sharing of water sources between humans and animals, drinking of untreated water, keeping large numbers of domestic animals, close interaction between humans, domestic animals and wildlife and a high proportion of HIV/AIDS patients (17-18%). The study districts are located in the cattle corridor in Uganda that stretches from the Northeast to the south-western region, occupying an area of about 40% of the total land and area, These are fragile ecosystems, receiving little rain (less than 1200 mm per annum), commonly hit by drought periods resulting in inadequacy of pastures and water [27]. Nakasongola district has two rainfall seasons every year, with the peaks between October to December and March to May, with moderate quantities of rain (875-1000 mm). The rainy seasons are divided by dry periods of about five months. The most common household water sources in the Nakasongola district are valley tanks and dams, while in Mubende the water resources are streams, boreholes, swamps and valley dams. In both study districts, large herds of animal populations and humans depend of these water sources for survival.

In each district two sub-counties were selected as study sites for sample collection and for the questionnaire survey. The sub-counties were Madudu and Kiyuni in Mubende and Nabiswera and Lwampanga in Nakasongola. Pastoral community homesteads where caseous lymphadenitis and tuberculosis related human cases had been reported [28] were purposively selected.

### Sample size determination of the questionnaire survey

Sample size determination was based on previous studies on prevalence and mortality associated with tuberculosis in HIV infected patients in rural Uganda [29]. In addition, NTM had been detected in cervical lymphadenitis in pastoral communities in the Karamoja region of Uganda [21]. The two studies showed a NTM prevalence of 2-7% (mean = 4.5%). Considering the population reports of the district state of environment National Environment Management Authority (NEMA) [24,25], and based upon the simple assumption that the study needed an absolute minimum of 10 households with NTM infection in the study population, we opted for 231 as a necessary minimum number of households, enabling also a reliable comparison between population factors at a prevalence of 10-20% [30].

### Study design and sampling strategy

The primary study unit was the household represented by an individual from each. The selection of study household was done from a list of households, based on a systematic sampling approach where every 5^th ^household on the list was chosen, and in collaboration with the community local leaders in the respective sub-counties. A total of 231 individuals (each a proxy for a household) were interviewed using standardised questionnaires.

The questionnaire included the following aspects; keeping of cattle or other animals, location of the night enclosures (kraals), sources of water used for humans and animals, type of water treatment used, type of animals seen at the water sources, degree of sharing water sources with domestic and wild animals, frequency of cleaning and replenishment of the household water receptacles.

The questionnaire was set up in English, translated to local languages (Runyankore, Ruruli, Runyoro or Luganda) before use, and retranslated to English. This process allowed standardisation of responses across the different ethnic groups that were involved. In order to ensure correct filling of the questionnaire forms and appropriate data compilation and entry, trained interviewers administered the questionnaire in the local languages to each participant.

### Collection of samples for mycobacterial analysis

Alongside the questionnaire survey, samples from water, soil and animal faeces were collected for mycobacterial detection to represent both the households and the environment around. Samples were collected the same day as the questionnaires were administered. Sample collection was conducted between September 2008 and January 2009 covering both dry and wet seasons of the year. Samples were collected from the pastoral environments in sterile containers, sealed and placed in a cool box with ice packs (4-10°C) and transported to the laboratory for storage at -8°C. A total of 310 samples were collected from the 231 households (Table 1). About 20% of the samples were collected in each of the following months; September, October, November, and December 2008 and January 2009.

**Table 1:** Samples from environmental sources in Uganda examined for mycobacteria

Sample type	Sample subtype	No. of samples	No. (%) of isolates
**Water**	Household drinking	130	17 (13.1)
	Valley dam	36	3 (8.3)
	Stream water	20	2 (10.0)

**Subtotal**		186	22 (11.8)

**Soil**	Pig shelter	25	5 (20.0)
	Cattle kraal	19	4 (21.1)
	Water source	47	14 (29.8)

**Subtotal**		91	23 (25.3)

**Animal faeces**	Pigs	18	1 (5.6)
	Cattle	13	2 (15.4)
	Wild birds	2	0 (0)

**Subtotal**		33	3 (9.1)

**Grand Total**		310	48 (15.5)

Soil around water sources, valley dams and streams with high human and animal activity, cattle night enclosures (kraals) and soils where pigs were tethered were included in this study. Surface layer soil was not collected as this had been subjected to solar radiation. Approximately 5-20 cm was moved below the soil surface at each marked spot before collecting approximately 2 g of the soil sample. Most of the animal enclosures and pig tethering spots are located at a distance less than 20 m from the pastoral household. Soil around water sources were collected near the water edge from points accessed by the communities and animals during water collection or watering process, these spots could be up to 1-2 km from the household. A total of 91 soil samples were collected (Table 1).

Thirty millilitres of drinking water was collected from valley dams, streams and ponds and at household level in the above selected households, using a sterile bottle. At household level, samples were also collected, and if biofilms or sediments were found in the water containers, these were included in the collected sample. Of the 231 households visited, 130 drinking water samples were collected. Each household water source and stream was visited once for water collection. A total of 9 valley dams were chosen based on the heavy dependence by communities and animal populations, and sampled once every fortnight in 5 months until the source was completely dried out. A total of 186 water samples were collected (Table 1).

Faecal samples from cattle, pigs and wild birds found around the water sources were collected. When encrusted, the surface of the faeces was scrapped off before sampling. A total of 33 faecal samples were collected (Table 1).

### Isolation and identification of mycobacteria

Culturing of samples was performed as described previously with slight modifications [31,32]. Approximately two gram of soil or faecal sample was transferred to a sterile tube and 4 ml physiological saline was added, followed by centrifugation. The water samples were centrifuged directly. All samples were decontaminated using 4% NaOH, followed by 5% oxalic acid with 0.1% malachite green and inoculated on four different media; Middlebrook 7H10 with and without antibiotics, Löwenstein-Jensen medium and Stonebrink medium (BD Diagnostics, Sparks, MD). The samples were incubated at 37°C for up to 8 weeks. Colony morphology was noted and Ziehl-Neelsen (ZN) positive colonies were further identified using the INNO-Lipa Mycobacteria v2 assay (Innogenetics, Ghent, Belgium), with the following modifications: The colonies suspended in TE buffer were pre-heated at 95°C for 25 minutes instead of 10 minutes and an extra extension step, 72°C for 10 minutes, was added after the 40 cycles in the PCR run. Isolates that could not be species identified by the INNO-Lipa assay were submitted to 16S rDNA sequencing, targeting the hypervariable region A (151 bp) [32,33]. The following primers were used: 16S8F (AGAGTTTGATCMTGGYTCAG) and 16SM259 (TTTCACGAACAACGCGACAA) [33-35]. Obtained sequences were edited and analyzed in Bioedit http://www.mbio.ncsu.edu/BioEdit/bioedit.html and sequences were compared to available sequences in GenBank by the NCBI Blast sequence alignment tool (National Centre for Biotechnology Information, http://blast.ncbi.nlm.nih.gov/). The isolate was determined to species based on the maximum score and maximum identity values on NCBI Blast alignment, a maximum score and maximum identity of ≥ 99% were accepted.

### Data Analysis

A database consisting of questionnaire data was established in Microsoft Excel^®^. Subsequent data from the laboratory work was added to the same database. Initial exploratory analyses were done in Excel using the radar chart to visualise the difference between households with slow growing (SGM) and rapid growing mycobacteria (RGM). The geographical location of households (Madudu, Kiyuni, Lwampanga and Nabiswera sub counties), keeping of cattle, drinking boiled or un-boiled water, sharing water sources with wild primates or antelopes and the nature of the water sources for household use (borehole, stream, and valley dams) were included in the radar chart. Further statistical analysis was carried out using Stata version 11/SE for Windows (StataCorp, College Station, TX). The outcome of interest for statistical modelling was any isolation of mycobacteria (SGM and RGM) in the environment of the households (i.e NTM presence in water, soil, and or animal faecal matter). The data analysed relates the actual presence of NTM (yes/no) to the documented exposures ascertained in the questionnaire. A multivariable logistic regression model was built using a backward selection strategy. The model fit was assessed using the Hosmer-Lemeshow goodness-of-fit test.

### Ethical approval

Ethical clearance was obtained from the Uganda National Council for Science and Technology (UNCST) and approved with a reference: H337.

## Results

**Figure 1:**
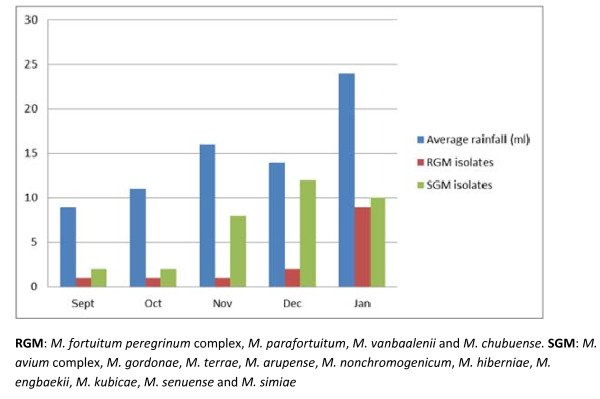
**Average rainfall in millilitres and number of non-tuberculous mycobacteria detected from environmental sources in Uganda during September 2008 to January 2009 **. Detection of rapid growing (RGM) and slow growing (SGM) mycobacteria are illustrated. RGM: *M. fortuitum peregrinum *complex, *M. parafortuitum*, *M. vanbaalenii *and *M. chubuense*. SGM: *M. avium *complex, *M. gordonae*, *M. terrae*, *M. arupense*, *M. nonchromogenicum*, *M. hiberniae*, *M. engbaekii*, *M. kubicae*, *M. senuense *and *M. simiae*.

Based on morphological characteristics and acid fastness by the ZN method, mycobacteria were detected in 48 (15.5%) of the 310 environmental samples. From the remaining 262 (84.5%) samples, no mycobacteria were detected; 108 samples did not show any growth of bacteria, while 154 samples showed massive overgrowth by other bacteria and fungi. Details on occurrence of various NTM in different samples are shown in Table 1 and Table 2, while temporal trends are illustrated in Table 3 and Figure 1.

**Table 2:** Rapid growing (RGM) and slow growing mycobacteria (SGM) isolated from different environmental sources in Uganda.

Category	Mycobacterial species				Sample source					Total
		Water			Soil			Faecal		
		**House** **hold**	**Valley** **dam**	**Stream**	**Pig** **shelter**	**Cattle** **kraal**	**Water** **source**	**Pig**	**Cattle**	

RGM	*M. fortuitum- peregrinum *complex	2	1		3	1	3	1	1	12
	*M. parafortuitum*				1					1
	*M. vanbaaleni*						1			1
	*M. chubuense*						1			1

**Total RGM**		**2**	**1**		**4**	**1**	**5**	**1**	**1**	**15**

SGM	*M. avium *complex	2			1	1	1			5
	*M. intracellulare*	1	1				7			9
	*M. gordonae*	4		1						5
	*M. terrae*	1								1
	*M. arupense*	1								1
	*M. nonchromogenicum*	3		1			1			5
	*M. hiberniae*					1				1
	*M. engbaekii*					1			1	2
	*M. kubicae*	2								2
	*M. senuense*		1							1
	*M. simiae*	1								1

**Total SGM**		**15**	**2**	**2**	**1**	**3**	**9**	**0**	**1**	**33**
**Grand total**		**17**	**3**	**2**	**5**	**4**	**14**	**1**	**2**	**48**
**%**		**35.4**	**6.3**	**4.2**	**10.4**	**8.3**	**29.2**	**2.1**	**4.2**	**100**

**Table 3:** Rapid growing (RGM) and slow growing mycobacteria (SGM) isolated from the environment in Uganda during September 2008 to January 2009.

Category	Mycobacterial species	Sept	Oct	Nov	Dec	Jan	Total
RGM	*M. fortuitum-peregrinum *complex	1	1	1	2	7	12
	*M. parafortuitum*					1	1
	*M. vanbaaleni*			1			1
	*M. chubuense*					1	1

**Total RGM**		**1**	**1**	**2**	**2**	**9**	**15**

SGM	*M. avium *complex		2	1	5	6	14
	*M. gordonae*	1	4				5
	*M. terrae*				1		1
	*M. arupense*			1			1
	*M. nonchromogenicum*			5			5
	*M. hiberniae*					1	1
	*M. engbaekii*					2	2
	*M. kubicae*				2		2
	*M. senuense*			1			1
	*M. simiae*	1					1

**Total SGM**		**2**	**6**	**8**	**8**	**9**	**33**

**Grand total**		**3**	**7**	**10**	**10**	**18**	**48**

Twenty percent of the samples were sampled each month.

Twenty-two mycobacterial isolates were detected from water, 23 from soil, and three from animal faeces (Table 1). Of these, 30 were identified by the INNO-Lipa test, and 18 samples were identified by 16S rDNA sequencing. Twelve isolates belonged to the *M. fortuitum-peregrinum *complex and 14 to the *M. avium *complex (MAC), while five were identified as *M. gordonae *and five as *M. nonchromogenicum*. Two isolates of *M. engbaekii *and *M. kubicae *and one isolate from eight additional mycobacterial species were also detected (Table 2). *M. gordonae*, *M. terrae, M. simiae*, *M. arupense, M. senuense *and *M. kubicae *were recovered only from the water environments, while *M. chubuense, M. hiberniae, M. vanbaalenii *and *M. parafortuitum *were recovered only from soil. *M. engbaekii *were detected in both soil and animal faecal samples. MAC and *M. nonchromogenicum *were recovered from water and soil and bacteria in the *M. fortuitum-peregrinum *complex was recovered from soil, water and animal faecal environments (Table 2).

In September only three isolates were recovered from the environment. In October, November, December and January, there was a steady increase in the amount of recovered NTM from the environments, from seven isolates in October to 18 isolates detected in January (Table 3 and Figure 1).

**Figure 2:**
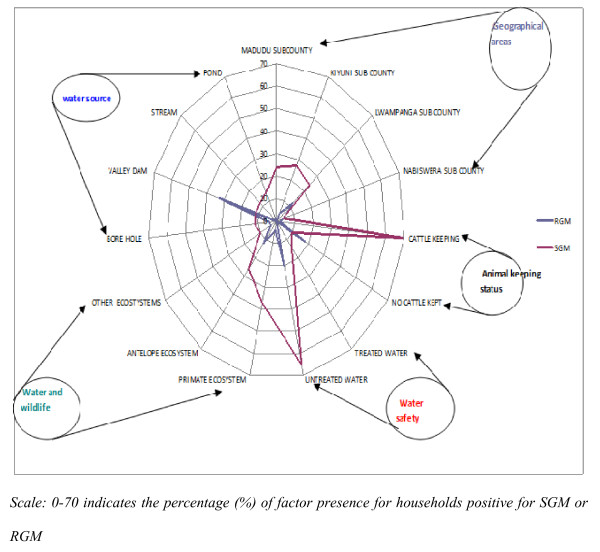
**A spider web presentation of the potential routes of exposure to environmental mycobacteria in the pastoral communities of Nakasongola and Mubende, Uganda **. Scale: 0-70 indicates the percentage (%) of factor presence for households positive for SGM or RGM.

The radar chart showed that drinking untreated water and having close contact with cattle or other domestic animals were common in households where slow growing mycobacteria (SGM) were found and drinking of water from the valley dams was the most pronounced factor related to rapidly growing mycobacteria (RGM) (Figure 2).

The multivariable logistic regression analysis confirmed these findings (Table 4). The dominant factor in the model was drinking untreated water (OR = 33). Therefore, this behaviour together with sharing of water sources with wild primates compared to antelopes (OR = 4.6), and sharing of water sources with domestic and wild animals (OR = 5.3) were the major practices associated with NTM presence in households. Drinking and domestic use of water from valley dams compared to streams (OR = 20) and closeness to cattle and other animals (OR = 13.8) were also important factors associated with the possibility of exposure to environmental mycobacteria. Assessment of model fit to observed data showed insignificant difference between the observed and predicted values (HL (χ^2^) = 5.47; P = 0.71).

**Table 4:** Multivariable logistic regression analysis examining factors associated with the likelihood of isolating non-tuberculous mycobacteria from the pastoral communities of Nakasongola and Mubende, Uganda.

Variable	Variable level	OR [CI, 95%]	P-value
Consumed untreated or treated water	Untreated water vs. treated water	33 [11.1-100]	0.0001
Close contact with cattle or other domestic animals	Yes vs. no	13.8 [3.8-19.0]	0.0001
Type of water sources used	Valley dams vs. spring	20 [14.3-25.0]	0.003
Shared water sources between animals and humans	Yes vs. no	5.3 [1.7-16.1]	0.003
Wild animals found drinking water at same water sources	Primates vs. antelopes	4.6 [1.3-17.0]	0.021

## Discussion

In the present study, a wide range of environmental mycobacteria from the human- animal- environment interface across the Ugandan rural pastoral farming ecosystem were detected. Furthermore, 15.5% of the 310 environmental samples collected contained mycobacteria. The yield of mycobacteria in the different samples could have been affected by several factors. They were collected in Uganda and transported to Norway for examination, something that delayed analysis. This could have greatly contributed to increased bacterial overgrowth by fungi and other bacteria. Ideally the samples size for water samples could have been bigger, but 30 ml was a convenient sample size for transportation. The decontamination method may affect the yield of mycobacteria, but is necessary to avoid growth of contaminants. The water samples contained a lot of organic material, containing other bacteria and fungi, and therefore the decontamination had to be the same as for soil and faecal samples, something that could have affected the number of isolates detected.

The results indicated that soils around the water sources were heavily contaminated, as mycobacteria were detected in 29.8% of the samples. This concurs with previous studies that detected MAC in 43% of soil and water samples in the environments around HIV patients in Uganda [36]. The high recovery of NTM in water and in soils around water sources might be linked to the runoff from carrying plant organic waste and animal waste, rich in humic and fulvic acids which in turn support the growth of NTM [37]. Thus animals and humans stand at risk of being infected or colonized by the opportunistic mycobacteria from the environment through collection and drinking of untreated water from these sources. Unfortunately this is a common practise in these areas [2,3,8,38].

Most of the isolates were detected in November, December and January (ten, ten and 19 isolates respectively). These months are in the last part of the rainy season, thus more isolates were recovered during the period that followed this season. This finding contradicts findings from Malawi [39], where higher recovery rates were found in the dry season samples compared to the wet seasons. The high contamination of limited water sources with faecal matter in the drier months could be playing a key role. Secondly surface evaporation from stagnant open water sources could also concentrate the level of organic matter contained in water source.

Humans can be infected/colonised with NTM without developing disease [13] and exposure to NTM in the environment is common. One of the major limitations of this study is the lack of accurate case diagnoses with disease in the communities sampled. As resources are limited, patients and animals presenting with granulomatous lesions in Uganda usually do not receive culture confirmation of the species involved. Therefore, the species of NTM isolated in the environment cannot be reliably linked to those causing disease in the patients exposed to them. However, the species isolated from the Ugandan ecosystems have been found to cause disease in patients in other parts of the world. For example bacteria in the *M. avium *complex are known to cause opportunistic infections in animals and humans [2], and have been isolated from cattle and humans with tuberculous lesions in Uganda [20,21], Zambia and other parts of the world [39,40]. *M. simiae *has been isolated from two Ugandan HIV infected patients [41]. The other NTM detected in this study have been isolated from patients in different parts of the world, and may cause clinical syndromes: chronic bronchopulmonary disease, lymphadenitis, skin and soft tissue disease, skeletal disease, and disseminated and catheter-related infections [39,40,42-46]. In humans, immunocompromised individuals such as the malnourished, HIV/AIDS infected, children or the elderly are often affected [47]. Given the high incidence of HIV/AIDS in Uganda, and especially in the districts of Mubende and Nakasongola, many patients might be at high risk of getting infected by NTM.

Bacteria in the *M. terrae *complex and *M. hiberniae *have been documented to be resistant to multiple anti-tuberculosis drugs [46]. *M. nonchromogenicum *and *M. terrae*, found to be non pathogenic for guinea pigs, pigs and rabbits are known to provoke a non specific hypersensitivity reaction to bovine tuberculin in guinea pigs, pigs and cattle [16].

Our findings describe the potential risk for humans and animals being infected with potentially pathogenic mycobacteria in the Ugandan rural farming communities. The questionnaire revealed factors of importance for human exposure to these NTM, as NTM were more often detected in water sources shared with domestic and wild animals (especially primates), and in valley dams. Close contact with cattle and other domestic animals, and drinking of untreated water were found to be important risk behaviour for possible exposure to NTM. This behaviour is common in the pastoral communities of Uganda, and in other rural communities in developing countries. Therefore, knowing that potentially pathogenic mycobacteria are present in the environment is important for identifying control measures for the human and animal population.

In a recent study from the same areas, pastoralists' knowledge was addressed. It appeared that people had generally little knowledge about the occurrence of mycobacterial infections, that these infections could be associated with the environment and wildlife and possible transmission from animals to humans [28]. Similarly, recent findings showed that wildlife and domestic animals had tuberculosis like lesions due to mixed infections of *Corynebacterium pseudotuberculosis *and NTM in Mubende, Uganda and South Africa [48,49]. These findings confirm that domestic and wild animals represent a permanent reservoir of mycobacterial infections and therefore pose a serious threat to control and elimination programs [50].

The present study suggests drinking untreated water, living in close contact with cattle or other domestic animals and drinking water from the valley dams as the most important factors for human exposure to NTM species. The importance of NTM should not be under rated in these pastoral communities where people live close to domestic and wild animals, and where they depend on contaminated water sources for household use. District reports indicate an increasing number of outbreaks of water-related diseases in humans due to increased contamination levels resulting from animals sharing water sources with humans [26]. Whether boiling of drinking water for human consumption could reduce the problems with NTM in drinking water is a subject of further research.

## Conclusions

The study detected a wide range of potentially pathogenic NTM in the environment around the pastoral communities in Uganda. Humans drinking untreated water and living in close contact with cattle or other domestic animals may be at risk of NTM infection. Further studies are required to assess to what extent humans and animals in these areas are infected by these environmental species.

## Competing interests

The authors declare that they have no competing interests.

## Authors' contributions

CK contributed to the design, data collection, laboratory work, drafting and writing of the manuscript. AM contributed to data collection, laboratory work, data analysis and drafting of the manuscript. BD contributed to conception, design, supervision and drafting of the manuscript. JOA and JO contributed to conception, design and supervision of the manuscript. MM contributed to design and drafting of the manuscript and VE contributed to the laboratory analysis and drafting of the manuscript. ES contributed to the acquisition of funds, design and drafting of the manuscript and TBJ contributed to the conception, design and laboratory work, supervision, drafting and writing of the manuscript. All authors have read and approved the final manuscript.

## Pre-publication history

The pre-publication history for this paper can be accessed here:

http://www.biomedcentral.com/1471-2458/11/320/prepub
